# Thermal Stresses in Maize: Effects and Management Strategies

**DOI:** 10.3390/plants10020293

**Published:** 2021-02-04

**Authors:** Muhammad Ahmed Waqas, Xiukang Wang, Syed Adeel Zafar, Mehmood Ali Noor, Hafiz Athar Hussain, Muhammad Azher Nawaz, Muhammad Farooq

**Affiliations:** 1 Institute of Environment and Sustainable Development in Agriculture, Chinese Academy of Agricultural Sciences, Beijing 100081, China; waqasclimate@hotmail.com (M.A.W.); atharhussainswu@yahoo.com (H.A.H.); 2 College of Life Sciences, Yan’an University, Yan’an 716000, China; 3 Department of Botany and Plant Sciences, University of California, Riverside, CA 92521, USA; 4 Key Laboratory of Crop Physiology and Ecology, Institute of Crop Science, Chinese Academy of Agricultural Sciences, Ministry of Agriculture, Beijing 100081, China; mehmood2017@gmail.com or; 5 Department of Horticulture, College of Agriculture, University of Sargodha, Sargodha 40100, Pakistan; Azher.nawaz@uos.edu.pk; 6 Department of Plant Sciences, College of Agricultural and Marine Sciences, Sultan Qaboos University, Al-Khoud 123, Oman; farooqcp@squ.edu.om; 7 Department of Agronomy, University of Agriculture, Faisalabad 38000, Pakistan

**Keywords:** climate change, heat stress, cold stress, maize, oxidative damage, tolerance/susceptibility, breeding and genomics, limitations in crop improvement

## Abstract

Climate change can decrease the global maize productivity and grain quality. Maize crop requires an optimal temperature for better harvest productivity. A suboptimal temperature at any critical stage for a prolonged duration can negatively affect the growth and yield formation processes. This review discusses the negative impact of temperature extremes (high and low temperatures) on the morpho-physiological, biochemical, and nutritional traits of the maize crop. High temperature stress limits pollen viability and silks receptivity, leading to a significant reduction in seed setting and grain yield. Likewise, severe alterations in growth rate, photosynthesis, dry matter accumulation, cellular membranes, and antioxidant enzyme activities under low temperature collectively limit maize productivity. We also discussed various strategies with practical examples to cope with temperature stresses, including cultural practices, exogenous protectants, breeding climate-smart crops, and molecular genomics approaches. We reviewed that identified quantitative trait loci (QTLs) and genes controlling high- and low temperature stress tolerance in maize could be introgressed into otherwise elite cultivars to develop stress-tolerant cultivars. Genome editing has become a key tool for developing climate-resilient crops. Moreover, challenges to maize crop improvement such as lack of adequate resources for breeding in poor countries, poor communication among the scientists of developing and developed countries, problems in germplasm exchange, and high cost of advanced high-throughput phenotyping systems are discussed. In the end, future perspectives for maize improvement are discussed, which briefly include new breeding technologies such as transgene-free clustered regularly interspaced short palindromic repeat (CRISPR)/CRISPR-associated (Cas)-mediated genome editing for thermo-stress tolerance in maize.

## 1. Introduction

Climate change is threatening food security across the globe [1]. Crop yield must increase by 25–70% by the year 2050 without putting pressure on ecosystem functioning [2,3]. Since the 1960s, the yield improvement rate of major food crops (rice, wheat, and maize) has slowed down [4], and current yield trends are not sufficient to meet future requirements [5]. Moreover, improvements in crop productivity must be attainable in a highly inconstant climate. More and intensified extreme climatic events (drought, heatwave, frost, heavy rainfall, storms, etc.) are anticipated in the future [6,7]. These unprecedented climatic extremes will negatively influence plant growth and development, ecosystem services, and human comfort [8].

Maize (*Zea mays* L.) crop provides 19.5% of global caloric intake from all sources [9,10]. Furthermore, it has also become an important industrial commodity. However, temperature extremes (occurrence of high and low temperatures during the growth period) are threatening the yield sustainability of maize. Maize plants are sensitive to heat stress (>30 °C) and there is a strong decline in grain yield as plants face heat stress above this threshold for a prolonged duration [11].

**Figure 1:**
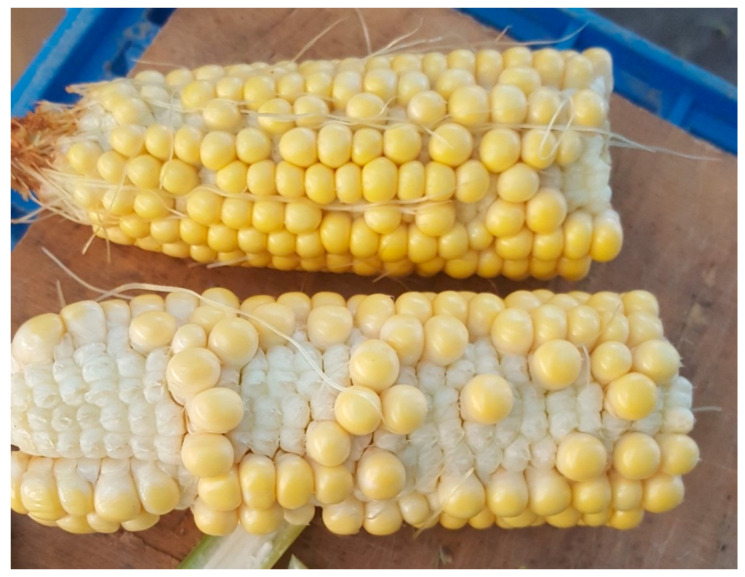
Reduced seed setting in summer maize (ZD958 sown in Hebei, China, in 2018) exposed to heat stress at the pollination stage. The temperature exceeded 35 °C at the time of pollen shed, affecting the pollen reception by initially emerged silks at the base of the ear as compared to the tip of the ear for late-emerging silks.

The optimal growth of maize crop needs different temperatures during day and night and over the whole growing season. During daylight, the optimal temperature varies from 25 to 33 °C, whereas during the night, optimal temperature varies from 17 to 23 °C; the mean optimal temperature for the whole growing season is 20–22 °C [12]. Maize plants germinate best at 25–28 °C [13]. The reproductive stage is the most sensitive to sub-optimal and supra-optimal temperatures. A swing from the optimal temperature causing high-temperature stress significantly decreases the growth rate and grain yield through a decrease in seed setting ratio and disturbance of several physiological processes (Figure 1). The minimum and maximum threshold temperatures at various growth and developmental stages of maize crop are presented in Table 1. It is projected that until 2050, 45% of the global maize production area is likely to face a mean episode of five days of the maximum temperature >35 °C during the reproductive stage annually [14]. This is important to note as a mere 1 °C rise in mean seasonal temperature can cut the economic yield of maize crop by 3–13% [15]. A high temperature at critical development stages may also deteriorate the quality of maize grains [16].

**Figure 2:**
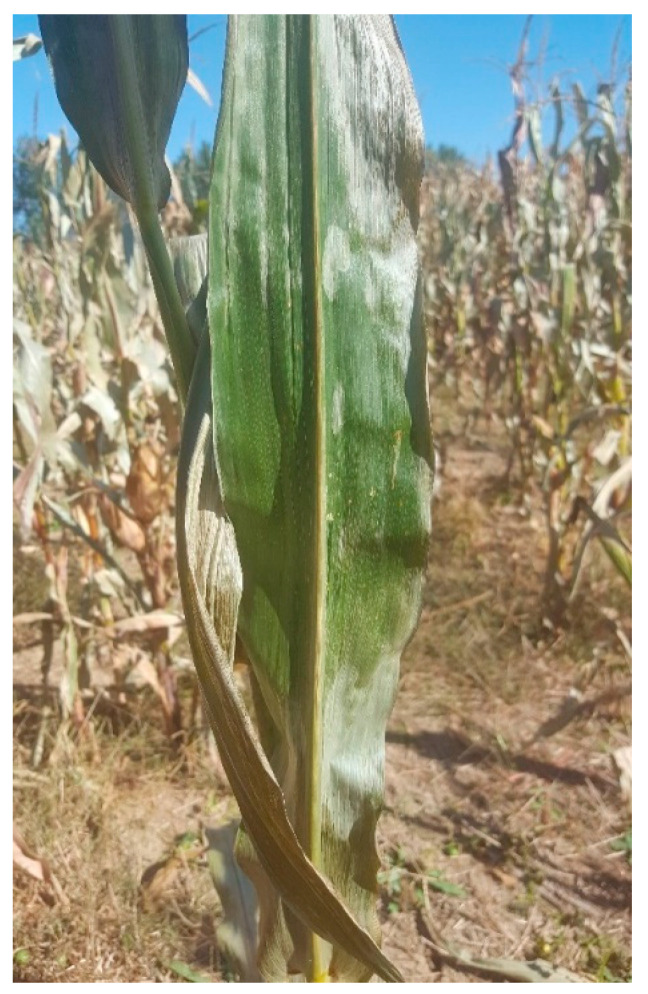
Chilling injury to maize plant after cold stress (XY335 sown in Hebei, China, in 2018). Leaf of maize plant affected by night cold wind of 2–4 °C, showing leaf wilting to leaf blade at the late reproductive stage.

Although maize crop is originally from the (sub-)tropics, its cultivation has moved to regions with temperate climates. Productivity loss under low temperature mainly occurs because of a strong decline in metabolite transport and photosynthetic activity [17] (Figure 2). In general, low temperature negatively affects gaseous exchange, water use efficiency, morphology, and physiology [10,18]. Farmers sow maize early to escape heat stress at the reproductive stage, but plants are exposed to low soil temperature (below 10 °C) during early seedling establishment. During this phase, soil temperature strongly impacts leaf development as the shoot apex is positioned very near to the soil surface. Therefore, to cope with temperature extremes in maize production, a comprehensive set of adjustments in cultural as well as in molecular techniques (such as breeding climate-resilient genotypes) and an improved understanding of the genetic, physiological, and molecular responses to temperature extremes are needed.

The published reviews focused either on heat or cold stress or on a particular stage of the crop [19,20]. Here, we review the impacts of both heat and cold stresses on maize production and elucidate recent developments in improving its performance. Besides, future research needs have also been highlighted.

## 2. Maize Growth under Temperature Extremes

Crop plants usually experience different biotic and abiotic stresses simultaneously that cause many morphological and physiological perturbations, resulting in stunted plant growth and reduced grain yields [21,22]. Temperatures above the threshold for various metabolic, biochemical, and physiological processes result in imbalance for these activities and activate the innate plant defense system [23]. Temperature extremes alter the photosynthetic process, damage the biological membranes, affect nutrient uptake, and limit the functioning of various enzymes in maize plants [24]. Stunted growth and low photosynthetic rates cause impairment in overall maize performance.

**Table 1:** Threshold temperatures for different growth stages in maize.

Growth Stages	Threshold Min. Temperature (°C)	Threshold Max. Temperature (°C)	Symptoms
Sowing–Emergence	10 ± 2.2	40 ± 2.1	Growth rate is severely decreased
Sowing–Tasseling	9 ± 2.7	39 ± 0.6	Abnormal tassel growth
Anthesis	8 ± 0.5	37 ± 1.4	Pollination failure may occur
Grain filling	8 ± 2.0	36 ± 1.4	Substantial decrease in starch and sucrose production
Whole maize crop	6 ± 1.1	42 ± 3.3	Low crop performance/Crop failure

Source: [25]. Data are mean ± standard errors of threshold temperatures for different growth stages in maize obtained from 70 publications using a meta-analytic approach. Threshold minimum temperature and threshold maximum temperature indicate the lower and upper thresholds at which tissue injury occurs or metabolic process may discontinue, respectively. At this temperature, maize yield loss can be sudden, huge, and irreversible.

### 2.1. High Temperature Impacts

**Agronomic**: Brief or prolonged episodes of high temperature stress during the maize growth cycle (especially at the most critical flowering stage) cause metabolic and/or morphological alterations, leading to irreversible yield reductions [25,26,27]. Maize leaf growth increases at temperatures ranging from 10 to 35 °C, while it starts declining at temperatures >35 °C [28]. Temperatures ranging from 33 to 36 °C during pre-and post-flowering regimes of maize, respectively, reduce the CO_2_ exchange rate (~17%), crop growth rate (17–29%), grain number (7–45%), and grain yield (10–45%) [29]. During flowering, high temperature negatively affects the floret number, silk number, and grain development [30]. Air temperature above 35 °C suppresses maize ovary fertilization and the grain filling process, which is directly associated with the final grain yield [16,31]. Heat stress decreases the time for tasseling and pollen shedding and enhances the anthesis-silking interval to reduce the viability and amount of pollens [31]. Moreover, heat-stressed maize plants are unable to convert photosynthates into starch in pollens. Thus, a decrease in pollen numbers, viability, and starch synthesis contribute to the distorting fertilization process [32]. Heat stress in tropical maize causes leaf firing, tassel blast and sterility, and senescence, leading to productivity loss [33].

**Figure 3:**
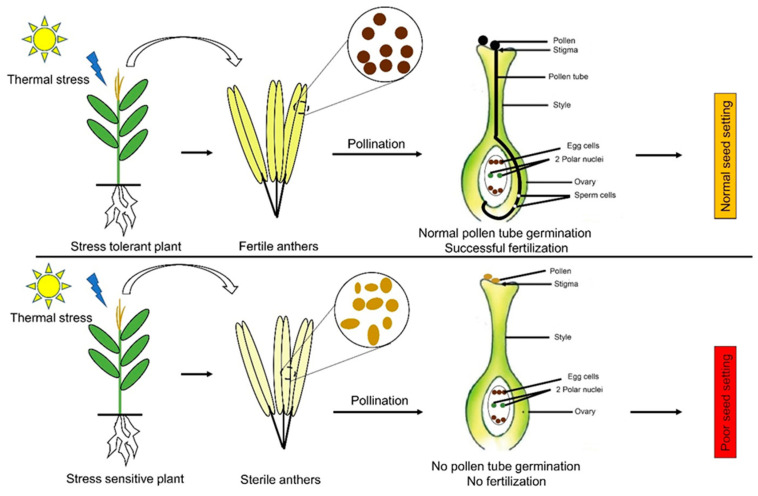
The influence of thermal stress on reproductive development in maize. Thermal stresses cause anther sterility and pollen abortion in the sensitive varieties, which lead to a lack of pollen tube growth and fertilization. This abnormality ultimately leads to a lack of seed setting in the maize cobs. On the other hand, tolerant varieties maintain their anthers and thus have viable pollens inside them, which leads to successful fertilization and normal seed setting.

Heat stress during the reproductive phase causes parchedness of silks, pollens’ sterility, and poor seed setting, resulting in drastic yield reduction [25,26]. Productivity loss at the reproductive phase due to heat stress is also linked with a decrease in the number of grains and their weight [34]. The day-time temperature of 35 °C in waxy maize reduced the grain yields by up to 31% due to decreased grain number and grain weight [35]. In response to heat stress stimuli, the defense mechanism of plants tends to opt to escape or avoid the stress period through phenotypic plasticity, which reduces the grain filling duration [34,36]. Interestingly, under elevated temperatures, the normal process of endosperm development is fully completed by the maize plant, but at a much accelerated pace. Furthermore, the maize plant only executes accelerated endosperm development under elevated day- and night-time temperature, not only under day-time warming [37], suggesting that heat stress impact varies greatly with the time of day and severity of stress. Figure 3 explains the influence of thermal stress on reproductive development in maize.

**Physio-metabolic:** Inadequate photosynthates in heat-stressed plants are often considered as a major limiting factor for yield [38]. Nevertheless, considering the C_4_ biochemistry of maize crop, usually, photosynthate supply is not limiting [39]. Thus, the primary cause of yield loss under elevated temperatures for the maize crop during the grain filling period is the accelerated developmental process. Grain yield decreases when yield formation operations are conducted earlier than normal. Key enzyme activities such as ADP-glucose pyrophosphorylase (involved in starch biosynthesis) are also limited at numerous levels, including both the transcriptional and post-transcriptional levels [37]. Notably, a high temperature at the grain filling phase reduces amyloplast biogenesis and endosperm cell division, causing a decrease in the grain size [27].

Starch accumulates in the developing grain through a complex network of enzymes (sucrose synthase, soluble starch synthase) regulating this pathway [40]. Heat stress limits these enzyme activities and impairs starch accumulation during the grain filling and hardening process [41]. High temperature (>30 °C) interrupts the normal amyloplast replication process and cell division in grains, thereby shrinking sink size [42]. Besides, high temperature affects the physicochemical properties (starch, protein, and soluble sugar contents) of waxy maize during the grain filling process, resulting in grains with substandard quality [43].

Heat stress also disturbs the normal physiological processes required for optimal maize growth and development. Reduced biomass assimilation and grain abortion are the key physiological processes resulting in reduced grain number in heat-stressed plants [30,44]. Heat stress up to 36 °C significantly decreased the radiation use efficiency [45], and less active nitrogen and carbon metabolisms contribute to a decrease in dry matter accumulation [46]. The discussion above suggests that radiation use efficiency, biomass accumulation, and the source–sink ratio are the crucial determinants of final grain yield and the harvest index under heat stress conditions. Taken together, heat stress negatively impacts pollens viability and silks’ receptivity, leading to a significant decrease in seed set and economic yield (Table 2).

### 2.2. Low Temperature Impacts

**Agronomic**: Maize is a cold-sensitive plant, and often, yield is limited in cool, humid regions (e.g., Central Europe). In these regions, when maize crop is exposed to cold stress, the growth rate tends to reduce while growth duration is prolonged. Thus, low temperature weakens the seedling and may also cease the grain filling prematurely at the end of the growth cycle [36,49,50], resulting in lower and inconsistent grain production in mountainous and temperate areas. Injury to plant cells or tissue under chilling stress during the early seedling stage or low temperatures at the reproductive stage in maize may vary depending upon the stress duration and its extent.

Low temperature stress, characterized by plant exposure to a temperature range below 10 °C for a sufficient duration, can interrupt the normal process of crop growth, starting from the early seedling stage to the later reproductive stages [51]. Chen et al. (2012) reported that low temperature in maize seedlings significantly limits germination and seedlings’ growth and destabilizes the antioxidant defense mechanism [52]. Cold stress negatively affects root morphology, photosystem II (PS II) efficiency, chlorophyll contents, and leaf area [53]. A short episode of low temperature stress (for instance, below 10 °C for 7 days) during the V6–V9 maize growth stages can significantly delay the anthesis initiation [54]. Among the morphological responses by stressed maize plants, low temperature stress causes abnormal tassel growth in maize [55], thus affecting the pollination and grain filling processes. Therefore, sub-optimal temperatures can cause a serious yield reduction if occurring at critical reproductive stages, as plants assign more than 50% of their photosynthates to develop grains during this phase until physiological maturity [56]. Low temperature stress significantly decreases the plant height and total crop biomass of maize [57]. Leaf development becomes slow in cold-stressed plants due to a prolonged cell cycle and decreased rate of mitosis [58].

**Physio-metabolic**: A temperature around 8–10 °C delays seedling emergence and causes a reduction in the root/shoot ratio and chlorophyll content during the early growth cycle in maize [59], whereas a temperature from 4 to 10 °C may suppress chlorophyll synthesis and causes a severe reduction in photosystem II (PS II) activity [60]. Low temperature stress negatively impacts chloroplast and thylakoid structures, enzyme activities, and the Calvin cycle by reducing metabolite transport [61]. While studying cell wall properties under chilling stress (12–14 °C), Bilska-Kos et al. (2017) reported that cell wall pectin content and pectin methylesterase activity become lower in a cold-sensitive maize hybrid [62]. Various physiological and biochemical disorders can be observed in photosynthetic machinery, cell membranes, and enzyme activities under low temperature stress [63]. Chen et al. (2012) reported a significant rise in malondialdehyde (MDA) contents and cell membrane permeability due to chilling injury at the early seedling stage, with reduced contents of water, proline, and chlorophyll in maize leaves [52]. Low temperature stress also makes shoots and roots macro-nutrient (N, P, K, Ca, Mg)-deficient by limiting metabolite transport [64]. However, when maize plants are exposed to chilling temperatures of 7–10 °C, they produce signaling compounds (e.g., nitric oxide and abscisic acid) in defense [65]. Low temperature stress causes damage to macromolecules, cellular structures, and membranes due to the excessive production of reactive oxygen species (ROS) [22,36,66]. In defense, plants produce more antioxidant enzymes including superoxide dismutase (SOD), peroxidase (POD), and proline [10,67].

**Figure 4:**
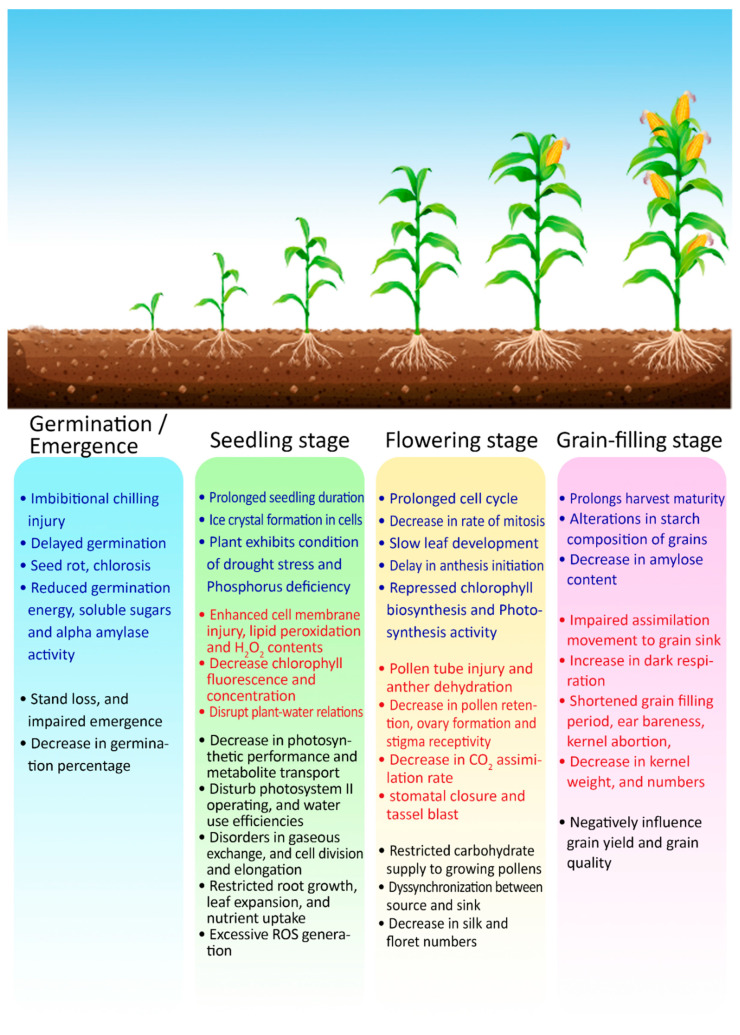
Overview of the impacts of cold and heat stresses on critical growth phases of maize crop. Heat stress impacts are marked by red fonts, while blue fonts indicate impacts of cold stress. Combined impacts (which can be caused by both stresses) are marked by black fonts.

Low temperature stress at grain filling can alter the starch composition in grains by reducing the amylose content, ultimately decreasing water solubility and starch swelling power and increasing gelatinization temperatures [43]. Temperatures below 15 °C during the late reproductive stage reduce the activities of the photosynthetic apparatus as well as rates of sucrose phosphate synthase, phosphoenolpyruvate carboxylase, and sucrose synthase. It tends to destabilize the assimilation process, resulting in impaired grain quality with substandard-quality components and poor physical grain texture [68]. Collectively, low temperature stress reduces the germination percentage, growth rate, and the photosynthetic rate, resulting in poor yield. A schematic representation of the various effects and mechanisms of heat and cold stresses are summarized in Figure 4.

## 3. Strategies to Mitigate the Effects of Temperature Fluctuations

To cope with the deleterious effects of temperature extremities, it is unavoidable to adopt multiple agronomic and breeding alternatives along with advanced genomic tools. Here, we discuss various strategies to combat temperature extremes in maize cropping systems.

### 3.1. Climate-Smart Agronomic Practices

Climate-smart agronomic practices for a specific cropping system include practices that help farmers adapt well to climate stresses and/or decrease productivity loss. These practices are becoming increasingly important to mitigate the adverse effects of temperature extremes [69,70]. Change in planting time may help plants escape the temperature extreme phase at critical growth stages [71]. In the North China Plain, maize crops have been confronted with episodes of chilling and heat stresses in recent years. Alteration in planting time helped reduce the yield losses significantly by minimizing the risk of heat and chilling damage during the silking and grain filling stages, respectively [34].

While switching to longer seasons, cultivars also enhanced the grain yield (ranging from 13% to 38%) by successfully mitigating the grave effects of increased warming trends of three decades [72]. In semi-arid areas (e.g., Sub-Saharan African countries), chances of maize crop failure are very high because of the harsh climate. In these areas, the technology of dry soil planting (DSP) is very effective to attain adequate grain yield [73]. Farmers sow seeds just before the rainy season in dry soil. Because seeds will be in soil at the time of rain, they can start the germination process instantly after receiving moisture. Such technologies can be strengthened with artificial intelligence more accurately predicting the rainy season [74]. Adoption of cultivars with more thermal time requirements can also significantly increase the yield by the delay in maturity and extended reproductive growth duration [75]. Therefore, farmers need to adapt to the future climate by optimizing the sowing date, maize earliness, and dry soil planting and selecting cultivars with more thermal time requirements according to their local pedoclimatic conditions.

### 3.2. Use of Plant Growth Regulators

Exogenous application of plant growth regulators (PGRs) such as thiourea, proline, salicylic acid, etc., can create tolerance against temperature extremes [18,76]. PGRs are modest in molecular weight and influence plant growth at very low concentrations. PGRs make plants’ defenses strong against external stress stimuli by scavenging ROS, making osmotic adjustments, stabilizing the integrity and structure of membranes and enzymes/proteins for normal functioning, inducing expression of antioxidant-related genes, and increasing the nutrient uptake as well as the biosynthesis of secondary metabolites and osmolytes [76]. Exogenous application of PGRs by seed priming (SP) and seed coating (SC) can significantly contribute to offsetting the adverse effects of temperature extremes on crop production. These practices enhance seed vigor, germination, physiology, and quality and provide uniform stand establishment under low and high temperature stresses [77,78]. Seed coating is an established technology to upgrade the seed performance under stress environments. Chemicals that regulate crop growth and development including insecticides, fungicides, plant growth stimulants, and fertilizers are used as seed coatings to guard seed against unfavorable conditions. Seed priming is a pre-sowing seed treatment that enables maize seeds to germinate fast [79]. Seed priming with both synthetic and natural growth promoters enhances maize performance under heat stress [24].

Low temperature in the area surrounding maize roots is a key problem in Northern and Central Europe which inhibits root growth and development, germination, early stand establishment, and nutrient uptake. Priming seeds with micronutrients (Zn, Fe, and Mn) improved the grain yield of maize by enhancing early seedling growth, germination, and nutrient uptake when maize seedlings were exposed to low soil temperature [80]. Seed priming with SA and H_2_O_2_ alleviated the negative impact of chilling tolerance by synergistically improving germination rate, seedling growth, α-amylase activity, energy supply, and antioxidant level (SOD, ascorbate peroxidase (APX), catalase (CAT), and glutathione reductase, GR) and upregulating the expression of their correspondence genes and genes involved in the biosynthesis of gibberellic acid ( ZmGA3ox2 and ZmGA20ox1), signaling (ZmGID1 and ZmGID2) and downregulating germination inhibition genes such as ZmRGL2 [81]. Likewise, SC technology is becoming popular worldwide, and every year, the proportion of maize plantations with coated seed is increasing [82]. SC with thermo-responsive hydrogel poly (N-isopropylacrylamide-co-butylmethacrylate) loaded with salicylic acid improved germination energy, root and shoot growth, and defensive enzyme production in the coated maize seeds to induce chilling tolerance [83]. SC with chitosan and hydrogen peroxide enhanced the emergence rate, protein content, and endogenous levels of H_2_O_2_ of maize seedlings [84]. The most yield-limiting factor under low temperature stress is the malfunctioning of photosynthetic machinery and metabolite transport. Exogenous treatment of S-Methylmethionine-Salicylate (a sulfur-containing compound that contributes to the methylation process and methionine biosynthesis) maintained high photosynthetic activity by enhancing defense gene expression in the phenylpropanoid pathway to protect maize plants under low temperatures [85]. Additionally, melatonin is a novel plant growth regulating substance that is widely accepted as a powerful antioxidant and signaling molecule. Melatonin application has improved heat and cold tolerance in various plant species [86,87]. This novel substance can also be utilized to improve the low and high temperature stress tolerance of maize varieties.

Plant growth regulators also offset the high temperature stress in maize production. SP with synthetic chemicals such as hydrogen peroxide, ascorbic acid, and salicylic acid improved maize morphological, physiological, and grain-related aspects (grain yield and grain quality) by improving cell membranes’ integrity, chlorophyll contents, antioxidants’ activity (CAT, POD, and SOD), and leaf water contents when plants were exposed to a high temperature at the reproductive and grain filling stages [88]. Foliar applications of natural (moringa fresh leave extract, sorghum water extract) and synthetic PGRs (salicylic acid, thiourea, ascorbic acid) were found effective in improving maize grain quantity and quality under thermal stresses by improving the growth, morphology, physiology, and antioxidant defense system of plants [18,88]. Although PGRs showed promising results to address the negative impacts of temperature extremes (Table 3), some caution is still required to be taken before their application. PGRs’ impacts are dependent on crop species, variety, applied dose, and even on application time (crop growth stage). Therefore, site-specific procedures suited to a place should be adopted by farmers with the advice of scientists.

### 3.3. Breeding for Thermal Tolerance

Among different approaches to cope with the deleterious effects of temperature stress, breeding tolerant cultivars is the most economical and sustainable one [93,94]. Climate-resilient cultivars can help increase the maize yield by 5–25% in Africa [95]. Huge variation exists among the maize germplasm for the degree of tolerance to temperature stress, which should be exploited [33]. Direct selection of germplasm based on higher yield performance under stress conditions is complicated [96]. Alternatively, breeding followed by selection based on secondary traits having a significant correlation with yield and its contributing traits is more effective and sustainable [33]. However, this selection should be based on cost-effective technologies. The use of sensors may also help accelerate plant breeding programs [97]. The integration of phenotyping with modeling can increase the selection effectiveness for stress tolerance based on complex traits [98].

High-throughput phenotyping (phenomics) has emerged as a novel tool of modern breeding that has tremendous scope for efficient selection. However, its use among breeders, particularly in developing countries, is still a challenge due to the relatively high cost, which should be addressed by international research organizations such as the International Maize and Wheat Improvement Center (CIMMYT) and the Food and Agriculture Organization (FAO).

Conventional breeding is a slow process and may take from several years to a decade to develop a new variety. Quick and shorter breeding cycles can help in developing new tolerant cultivars in less time [99]. Shuttle breeding, introduced by the CIMMYT, is another option to reduce the time required in developing a variety. This system can help to have an extra generation advanced each year at a different field location.

#### 3.3.1. Breeding for Heat Tolerance

Identification of suitable parents is vital for any breeding program, keeping in mind the objectives of the study. For instance, the selection of high-yielding heat-tolerant varieties to be used as parents is a pre-requisite to start a breeding program aimed at the development of high-temperature-tolerant maize cultivars. Here, we enlist several maize genotypes including inbred lines and hybrids that showed significant heat tolerance and, thus, can serve as important breeding materials to introgress heat tolerance in elite maize cultivars (Table 4). Similarly, the identification of key selection indices is crucial for the selection of tolerant cultivars or wild species. Leaf firing, tassel blast, tassel sterility (TS), anthesis-silking interval (ASI), and senescence are negatively correlated, while pollen shedding duration (PSD), seed setting percentage (SSP), and chlorophyll content are positively correlated indices with grain yield in maize under heat stress [33]. Recently, it was found that high temperature affects the carbon dioxide exchange rate (CER) in maize, which negatively affects crop growth rate, grain number, and final grain yield [29]. Photosynthesis fitness is critical in deciding the performance of maize crops under heat stress conditions [8]. Sustaining a satisfactory rate of photosynthesis activity under heat stress is essential to reduce productivity loss [100,101]. Several indicators of photosynthesis fitness have been reported such as chlorophyll contents, carotenoids, and stay-green plant architecture which are positively correlated with the rate of photosynthesis [102]. Normalized difference vegetative index (NDVI), based on the characteristic reflectance features of maize canopy, is an efficient indicator of the stay-green trait [103]. Thus, employing these traits in breeding programs of heat-tolerant high-yielding maize cultivars can increase reproductive success, photosynthesis efficiency (NDVI), and other yield-related traits under heat stress.

**Figure 5:**
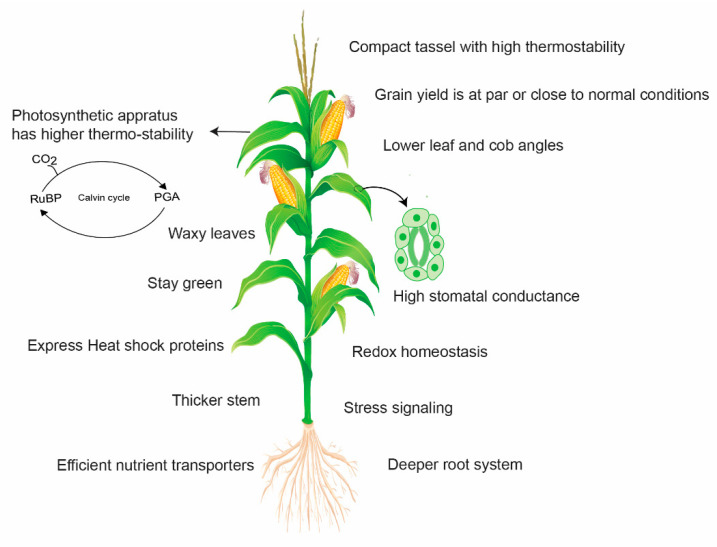
A simplified pattern representing the potential mechanisms involved in yield loss avoidance by heat-tolerant maize. Tolerant maize genotypes activate stress signaling and heat shock proteins (HSPs) and maintain redox homeostasis. HSPs improve protein and thylakoid stability and carbon assimilation to maintain high photosynthetic activity under heat stress. Leaves and tassels are more thermostable to avoid leaf firing and tassel blast, respectively. Lower leaf angle enhances radiation use efficiency by avoiding light saturation and increasing light penetration into the canopy. It also contributes to the quick cooling of leaves after exposure to heat. Waxy leaves guarantee a satisfactory water status in plants and higher water use efficiency. Thicker stem accumulates more carbon reserves for ear and kernel development. Heat-tolerant maize also has a deep rooting system to acquire scarce resources such as water and nutrients under stress environments.

Wild relatives and distant parents in intra-specific crosses are very useful resources to introgress novel genes for maize improvement. Teosinte, a progenitor of cultivated maize, harbors a lot of worthy genes to tolerate a combination of different stresses. Teosinte is well adapted to the high temperature environment as it shows relatively lower damage and sustains chlorophyll content under heat stress (36 to 45 °C) and depicts higher survival capacity even at 55 °C [104]. Therefore, it can serve as a potential source for maize improvement programs. However, it was poorly exploited for the identification and introgression of such genes. In the past, an effort was made to identify a heat-tolerant variety of teosinte called “Florida” and successfully introgressed heat tolerance from teosinte to cultivated corn [105]. According to another report, inter-subspecific hybrids of teosinte × maize were developed, which showed increased thermo-tolerance for several growth- and yield-related traits [104]. Thus, the exploitation of wild relatives and distant parents in intra-specific crosses could prove a very useful resource to introgress novel genes for maize improvement. Figure 5 indicates the potential mechanisms involved in yield loss avoidance by heat-tolerant maize.

#### 3.3.2. Breeding for Cold Tolerance

The maize crop is quite sensitive to low temperatures and requires fairly high temperatures for optimum growth and production. To avoid frequent episodes of heat and drought during the reproductive phase, farmers grow this crop early [24,106]. However, early grown maize is often exposed to chilling stress, which may lead to low crop performance due to poor germination or lack of seedling survival [107]. Sweetcorn is even more sensitive to low temperatures compared with field maize. It is imperative to attain high emergence percentages and vigorous seedlings under low temperatures to adapt maize for early sowing [108]. Massive variation is present in maize germplasm for adaptation to cold tolerance, especially in exotic maize populations [109]. Maize cultivars of temperate regions (e.g., Europe) have been widely used in chilling tolerance breeding programs based on good crop performance [60]. Here, we present several cold-tolerant maize cultivars developed around the world that could be utilized in breeding programs (Table 4). Mid-parent performance is a poor indicator of hybrid selection for cold tolerance, and testcross performance should be used as a reliable indicator for quantitative trait locus (QTL) mapping to develop stable markers [110]. Identification of reliable selection indices for cold tolerance is important to screen germplasm for the breeding programs [22]. Several traits such as photosynthetic rate, stomatal conductance, quantum efficiency, dry matter production, leaf weight and area, and water use efficiency are good selection indices to realize cold tolerance in maize [50]. Thus, the identification of cold-tolerant germplasm based on reliable selection indices can efficiently improve performance.

**Table 4:** Cold-/heat-tolerant maize genotypes developed using conventional breeding methods.

Inbred Line/Hybrid	Trait	Stress	Crop Region	Reference
Parents: B76, Tx205 Inbred lines: C273A, BR1, B105C, C32B, S1W, and C2A554-4	Low leaf firing and tassel blast	Heat tolerance	Texas, USA	[52]
Hybrids: YH-1898, KJ. Surabhi, FH-793, ND-6339, NK-64017	Improved grain yield	Heat tolerance	Punjab, Pakistan	[111]
ZPBL 1304	Heat shock protein	Heat tolerance	South Dakota, USA	[112]
Howling Mob	Emergence and shootrRoot dry weight	Cold tolerance	Wisconsin, USA	[107]
EP80 x Puenteareas	Emergence	Cold tolerance	Spain	[113]
Hybrids: AR1262, DKC6697, DKC6804, and M2V707	Leaf and root weights and root length	Cold tolerance	Mississippi, USA	[106]
Papirika	Relative tassel length	Cold tolerance	Hokkaido, Japan	[54]

### 3.4. Molecular Approaches

Genetically modified crops (GMCs) could serve as a useful resource for novel traits [114,115]. In recent decades, rapid progress in plant molecular biology has accelerated the rate of crop improvement. Several approaches including quantitative trait locus (QTL) mapping, transcriptomics, marker-assisted selection (MAS), map-based gene cloning, and genome editing (such as clustered regularly interspaced short palindromic repeat ((CRISPR)/CRISPR-associated-9, Cas9) have been utilized for selection and improvement of plant traits in several crops.

#### 3.4.1. Marker-Assisted Selection (MAS)

Pyramiding useful genes followed by the selection of desirable plant material has been a challenge for plant breeders. It is nearly impossible to pyramid multiple desirable genes through conventional breeding due to linkage. Marker-assisted selection (MAS) significantly improved the efficiency as well as decreased the time needed for complex trait selection such as drought, salt, cold, and heat tolerance [116]. Multiple genes control heat tolerance traits in maize crops. After the discovery of numerous molecular markers for cold and heat tolerance in maize, it is now possible to screen the tolerant germplasm at the early growth stage, saving time, labor, and space [46,117]. Single nucleotide polymorphisms (SNPs) are commonly used molecular markers due to their abundance in the genome, easy detection analysis, and co-dominance nature [8]. Several SNPs associated with traits governing heat and cold tolerance were identified, which could be employed in MAS to accelerate the selection process and speed up overall breeding programs [35].

#### 3.4.2. QTL Mapping for Candidate Genes

The majority of complex plant traits (for instance, tolerance to temperature extremes) are controlled by multiple genes, also called QTLs. The QTL may be a major or minor effect, depending on its influence on the concerned plant trait. Major-effect QTLs are more influential due to their substantial contribution to the expression of plant traits. Studies have reported major and minor effects of QTLs related to cold and heat tolerance in maize [118,119,120]. However, before the application of these QTLs in molecular breeding, it is imperative to confirm these QTLs in subsequent populations to validate their presence in wide germplasm [121]. Although, little has been done to explore the QTLs for heat tolerance compared with cold tolerance in maize. A recent study identified 11 QTLs including two QTLs for grain yield for heat tolerance in maize [118]. These QTLs were identified considering multiple environments and populations to increase the reliability to be used in breeding programs [118]. Six significant QTLs were identified among two large maize inbred panels for cold tolerance considering flint and dent traits [122]. QTL mapping with seedling-based F2:3 populations revealed seven QTLs for chilling tolerance in maize [123]. Four significant-effect QTLs related to cold tolerance of the photosynthetic apparatus were identified on chromosomes 1, 2, 3, and 9 and suggested the involvement of key genes on chromosome 3 in the development of functional chloroplast in maize [120]. Since good germination is a vital trait in cold stress environments, it is necessary to breed high yielding cultivars for better germination ability under low temperatures. Six QTLs controlling germination rate under low temperatures were detected on chromosomes 4, 5, 6, 7, and 9, and a single QTL explained contribution rate between 3.39% and 11.29% [124]. Seedling vigor is an important trait that can provide fast and uniform germination, vigorous crop stand, and stress tolerance. Five meta-QTLs were identified for maize seed vigor and were suggested for inclusion in cold tolerance breeding programs [108]. The introgression of multiple QTLs in elite maize germplasm can pave the way to attain temperature tolerance at multiple growth stages.

QTL mapping or genome-wide association studies (GWASs) followed by candidate gene analysis is a very useful approach to identify potential genes related to target traits such as heat or cold tolerance. However, validation of these candidate genes using other approaches such as over-expression or real-time expression analysis is important before utilization in breeding programs. Here, we present a list of potential genes linked with heat and cold stress tolerance that have been identified and validated using multiple approaches or multiple populations (Table 5). These genes could serve as a useful genetic resource in breeding climate-resilient maize cultivars.

#### 3.4.3. Transcriptomics

Availability of the complete genome sequence made it possible to explore the hidden genetic potential of maize crop. During recent decades, the advent of microarray-based transcriptome played a significant role in identifying plenty of desirable genes with comparatively less effort. Currently, the development of RNA-Seq-based whole transcriptome further fast-tracked the identification of key genes involved in complex traits such as cold and heat tolerance [130]. A recent comparative transcriptomic profiling identified 516 upregulated and 1261 downregulated genes among heat-tolerant (Xiantian 5) and heat-sensitive (Zhefengtian) maize varieties, which offers novel insights into the underlying molecular mechanisms of maize under heat stress [131]. Further quantitative real-time PCR (qRT-PCR) analysis discovered that five genes linked with secondary metabolites’ biosynthesis and photosynthesis have higher expression in Xiantian 5 compared with Zhefengtian, suggesting their role in heat tolerance in maize by improving photosynthesis and secondary metabolites’ biosynthesis. It was further explained that heat-tolerant sweet maize regulates heat stress responses by downregulating the expression of genes related to zeatin and brassinosteroid biosynthesis [131]. Microarray-based transcriptome analysis of cold-tolerant (ETH-DH7) and cold-sensitive (ETH-DL3) varieties identified the potential genes encoding cell membrane/wall proteins, playing a key role in cold tolerance possibly by protecting cell membrane/wall from the damaging effects of chilling injury [132]. Recently, RNA-Seq-based whole-transcriptome profiling reported 948 differentially expressed genes among cold-tolerant and -sensitive varieties under freezing stress of −1 °C [133]. These genes were involved in binding functions, protein kinase activity, and peptidase activity. The qRT-PCR of the 30 selected genes further validated the RNA-Seq findings and provided a new valuable resource for target breeding. These genes should be functionally characterized to be exploited in breeding for low temperature tolerance [133]. More recently, a microarray-based transcriptome profiling of four maize varieties having contrasting cold tolerances for seedling emergence identified 64 differentially expressed genes; among them, 11 were reported to be most significantly involved in seedling cold tolerance [134]. Thus, these transcriptome-based identified genes need to be functionally characterized to better understand the physiological and molecular mechanisms of cold tolerance [133].

#### 3.4.4. Map-Based Cloning

Map-based cloning is a widely used molecular biology tool to functionally characterize target genes. This approach usually requires gene mapping using mapping populations or recombinant inbred lines followed by cloning and complementation [135]. Several genes related to heat and cold tolerance in maize have been mapped followed by cloning. For instance, the genes *AOX* and *Zm-AN13* regulate germination at low temperatures [136]. *ZmCCT* and *ZmCCA1* contribute to stress tolerance in maize crop [114]. Another gene, *ZmLEA3*, protects lactate dehydrogenase activity to provide cold tolerance [133]. Sec14-like proteins regulate crucial biological processes, e.g., stress signaling, phospholipid metabolism, and stress response [137]. Overexpression of ZmSEC14p (Sec14-like protein) of maize plants conferred cold tolerance in transgenic *Arabidopsis*, suggesting that the *ZmSEC14p* gene can help plants to develop cold tolerance [138]. These identified and functionally characterized genes should be employed in gene introgression programs to induce cold and heat tolerance in maize cultivars.

#### 3.4.5. Genome Editing

Among all molecular breeding approaches, genome editing is quite a new breeding tool, especially CRISPR-Cas9. It is a powerful instrument to target desired genes with high precision [139]. Being less laborious and relatively easy, CRISPR-Cas9 is becoming increasingly important to understand various molecular mechanisms and characterize gene functions [140]. Although CRISPR-Cas9 has been used mostly for the development of disease and insect resistance in plants [141,142], it is relatively less applied for abiotic stress tolerance [139]. However, recently, genome editing for drought tolerance was successfully obtained in maize by CRISPR-Cas9 [143]. Researchers developed *ARGOS8* lines by replacing the native promoter of the *ARGOS8* gene (a negative regulator of ethylene responses) [144] with the maize plants’ GOS2 promoter at the 5′ untranslated region [143]. Highly precise targeting of the maize native promoter by CRISPR-Cas9 increased *ARGOS8* expression levels and improved grain yield under drought conditions [143]. CRISPR-Cas9-mediated heat tolerance was attained by targeting *SlAGAMOUS-LIKE 6* (*SIAGL6*) [145]. Likewise, CRISPR-Cas9 can develop heat- and cold-tolerant maize cultivars via genome editing of target genes [139].

Notably, transgene-free genome editing was successfully practiced in several major crops, which presents a remarkable strategy for targeted crop improvement [139,142,146]. Several techniques of transgene-free genome editing were introduced in different crop species such as protoplast-based plant regeneration in *Arabidopsis*, rice, tobacco, etc. [146], bombarding immature embryos in maize [147], and a more efficient callus-based transformation system in wheat [148]. These discoveries offer a great opportunity for rapid crop improvement with precise genome editing in a transgene-free manner.

## 4. Limitations in Crop Improvement

Maize cropping systems’ sustainability is threatened globally by unexpected shocks of more frequent temperature extremes during the growth period. Although maize breeders are working to improve crop performance, breeders from poor countries do not have access to major germplasm resources such as CIMMYT and International Center for Agricultural Research in Dry Areas (ICARDA) like developed countries do. The lack of a comprehensive communication system and coordination among breeders for sharing germplasm and knowledge aggravates the problem. Recently, the emergence of high-throughput phenotyping and genotyping facilitated maize breeders in the identification of efficient selection indices and molecular markers, respectively, leading to effective germplasm screening for target traits. Nevertheless, high-throughput phenotyping and genotyping facilities are not widespread among maize breeders of poor countries due to restricted funds, detectable by the slow rate of crop improvement in these areas [149]. This causes a huge difference in the average yield of maize between developed and under-developed countries [150]. For example, the average maize yield in the United States is 13.2 tons/ha, which is 340% more than the average grain yield of 3 tons/ha in South Africa [151] and 0.9 tons/ha in Mozambique [152]. Another key limitation is inadequate knowledge of the molecular mechanisms of complex traits such as heat and cold tolerance. Lack of appropriate infrastructure, inadequate operational support, limited human resources, and lack of enabling policies and statutory and regulatory frameworks are the key factors that hamper the prosperous growth of molecular breeding in developing countries [153]. Furthermore, poor communication of breeders with molecular biologists slows down maize improvement programs worldwide. A serious effort is necessary to address these key challenges among maize breeders across the world to ensure sustainable maize production and food security. This could be achieved through funded training of maize breeders from developing countries at international research stations such as CIMMYT, ICARDA, etc., to develop an excellent human resource for mid-economy countries. Another key step could be to provide instruments for high-throughput phenotyping and genotyping as well as seeds of improved cultivars to these breeders, which can boost their yield potential.

Recently, a lot of transcriptome studies have been performed in maize, just like in other crop species, to identify key genes for stress tolerance. However, studies leading to the functional validation of identified differentially expressed genes (DEGs) are lacking in maize, which is another reason for the slow rate of maize improvement. The reason behind this is the difficulty in the selection of candidate genes for functional validation due to a large set of genes. This could be addressed by using modern statistical tools to study the association of key DEGs with target traits that will refine the search for key candidate genes, as described in these studies [154,155].

## 5. Conclusions

Thermal stresses at critical growth stages of maize reduce the grain yield, nutritional value, and net income of farmers. Genetic variation exists among different cultivars for cold and heat tolerance, which indicates the need for more systematic plant breeding programs to have site-specific plant resources to improve maize crop performance under limiting growing conditions. Exogenous use of synthetic and natural plant growth regulators at low concentrations also reduces productivity loss under such circumstances. New breeding techniques such as marker-assisted breeding and genome editing, particularly the transgene-free CRISPR-Cas9 system, offer great potential for the development of climate-resilient cultivars in a comparatively shorter time. Additionally, a strong infrastructure for evaluation of maize germplasm based on high-throughput phenotyping plus genotyping is required in developing countries.

## Figures and Tables

**Table 2:** The impacts of heat stress on maize grain yield.

Growth Stage	Temperature	Yield Reduction (%)	Country/Region	References
Silking	6 °C above ambient canopy temperature for 3 days	13%	USA	[16]
Grain filling	35 °C	31%	China	[35]
Pre- and post-flowering	33 to 36 °C	10–45%	Argentina	[29]
Tasseling stage	30–38 °C for 15 days	14–17%	China	[24]
Grain filling	28–32 °C	10%	US corn belt	[47]
Reproductive stages	Each degree above 30 °C	1–1.7%	Africa	[48]

**Table 3:** Use of plant growth regulators to minimize the impacts of heat and cold stresses at critical phases of maize crop.

Strategy	Plant Growth Regulators	Concentration	Suboptimal Temperature and Growth Stage	Indicators of Stress Alleviation	Increment in Yield (%)	Reference
Seed priming	Fe	8.5 mM	Chilling (12 °C) stress at seedling stage	Better early seedling growth, germination, and nutrient uptake	13	[80]
Seed priming	SA	20 mg L^−1^	Chilling (10 °C) stress at seedling stage	Improved antioxidant enzymatic activities, water status, chlorophyll contents, and membranes structure	25	[89]
Seed priming	CaCl_2_	2.2%	Chilling (12 °C) stress at seedling stage	Improved leaf expansion; net assimilation	12	[90]
Seed priming	Kinetin	100 mg L^−1^	Heat stress (38 °C) at reproductive stage	improved stand establishment and phenolic contents and increased leaf area expansion and grain filling period	18	[91]
Magnetic seed stimulation	-	150 mT for three minutes	Chilling stress (≤10 °C) at stand establishment	Improved chlorophyll, phenolics, and gaseous exchange attributes	20	[92]
Foliar application	Thiourea	0.1%	Chilling stress (≤12 °C) at reproductive stage	Improved crop growth rate, water use efficiency, photosynthetic rate, and dry matter accumulation	21	[18]
Foliar application	Moringa leaf extract	3%	Heat stress (38 °C) at reproductive stage	Increase in leaf expansion and grain filling duration	17	[91]
Foliar application	AsA	20 mg L^−1^	Heat stress (38 °C) at reproductive stage	Increase in antioxidant activities and membrane stability	23	[88]
Foliar application	H_2_O_2_	20 mg L^−1^	Heat stress (38 °C) at reproductive stage	Enhanced SOD, CAT, and POD activities and grain weight	23	[88]

**Table 5:** Potential genes related to temperature stress tolerance in maize.

Gene	Target Trait	Effect of Gene	Approach Used to Characterize Gene Function	Reference
ZmDHN13	Abiotic stresses	Oxidative balance	Over-expression	[125]
ZmWRKY106	Heat tolerance	Reactive oxygen species (ROS) scavenging	Over-expression	[126]
ZmERD3	Heat and cold	mRNA accumulation	qRT-PCR	[127]
ZmbZIP4	Abiotic stresses	Abscisic acid (ABA) synthesis	Immunoprecipitation sequencing	[128]
GRMZM2G377194	Thermo-tolerance	Increased seed set	Quantitative trait locus (QTL) mapping plus genome-wide association studies (GWAS)	[129]
GRMZM2G060349	Thermo-tolerance	Increased seed set	QTL mapping plus GWAS	[129]
GRMZM2G122199	Thermo-tolerance	Increased seed set	QTL mapping plus GWAS	[129]
GRMZM2G026892	Thermo-tolerance	Increased seed set	QTL mapping plus GWAS	[129]
GRMZM2G148998	Heat tolerance	High grain yield	QTL mapping and potential gene analysis	[118]
GRMZM2G115658	Heat tolerance	High grain yield	QTL mapping and potential gene analysis	[118]
GRMZM2G537291	Heat tolerance	High grain yield	QTL mapping and potential gene analysis	[118]
GRMZM2G324886	Heat tolerance	High grain yield	QTL mapping and potential gene analysis	[118]
GRMZM2G436710	Heat tolerance	High grain yield	QTL mapping and potential gene analysis	[118]
GRMZM2G094990	Heat tolerance	High grain yield	QTL mapping and potential gene analysis	[118]
GRMZM2G178486	Cold tolerance	Improved germination	Gene cloning plus GWAS	[124]
GRMZM5G806387	Cold tolerance	Improved germination	Gene cloning plus GWAS	[124]
GRMZM2G148793	Cold tolerance	Improved germination	Gene cloning plus GWAS	[124]

## Data Availability

Not applicable.
